# Acclimation of *Nodularia spumigena* CCY9414 to inorganic phosphate limitation – Identification of the P-limitation stimulon *via* RNA-seq

**DOI:** 10.3389/fmicb.2022.1082763

**Published:** 2023-01-04

**Authors:** Mariano Santoro, Christiane Hassenrück, Matthias Labrenz, Martin Hagemann

**Affiliations:** ^1^Department of Biological Oceanography, Leibniz Institute for Baltic Sea Research, Warnemünde (IOW), Rostock, Germany; ^2^Department of Plant Physiology, Institute for Biosciences, University of Rostock, Rostock, Germany

**Keywords:** alkaline phosphatase, cyanobacterial bloom, diazotroph, polyphosphate, toxin, transcriptomics, transport

## Abstract

*Nodularia spumigena* is a toxic, filamentous cyanobacterium capable of fixing atmospheric N_2_, which is often dominating cyanobacterial bloom events in the Baltic Sea and other brackish water systems worldwide. Increasing phosphate limitation has been considered as one environmental factor promoting cyanobacterial mass developments. In the present study, we analyzed the response of *N. spumigena* strain CCY9414 toward strong phosphate limitation. Growth of the strain was diminished under P-deplete conditions; however, filaments contained more polyphosphate under P-deplete compared to P-replete conditions. Using RNA-seq, gene expression was compared in *N. spumigena* CCY9414 after 7 and 14 days in P-deplete and P-replete conditions, respectively. After 7 days, 112 genes were significantly up-regulated in P-deplete filaments, among them was a high proportion of genes encoding proteins related to P-homeostasis such as transport systems for different P species. Many of these genes became also up-regulated after 14 days compared to 7 days in filaments grown under P-replete conditions, which was consistent with the almost complete consumption of dissolved P in these cultures after 14 days. In addition to genes directly related to P starvation, genes encoding proteins for bioactive compound synthesis, gas vesicles formation, or sugar catabolism were stimulated under P-deplete conditions. Collectively, our data describe an experimentally validated P-stimulon in *N. spumigena* CCY9414 and provide the indication that severe P limitation could indeed support bloom formation by this filamentous strain.

## 1. Introduction

Mass developments, so-called blooms, of toxic cyanobacteria occur worldwide in freshwater or coastal brackish water systems and are of increasing concern, as they negatively impact the use of water for drinking and/or for recreation purposes. Global warming and climate change scenarios are expected to increase the frequency of bloom events in the next years (Paerl and Huisman, 2008). Cyanobacterial blooms are usually dominated by toxic colony-forming *Microcystis* spp. strains in freshwater. Filamentous cyanobacteria capable of atmospheric N_2_ fixation in heterocysts are frequently occurring in brackish water blooms, where *Nodularia spumigena* is often dominating. This filamentous cyanobacterium can produce the potent hepatotoxin nodularin and occurs worldwide in coastal waters (Sivonen et al., 1989; Gehringer and Wannicke, 2014).

Key factors favoring growth and bloom formation of N_2_-fixing, filamentous cyanobacteria in the Baltic Sea include the availability of phosphorus (P) sources in combination with low to undetectable combined nitrogen concentrations (Sellner, 1997). The virtual absence of combined nitrogen sources after the diatom spring bloom promotes the dominance of N_2_-fixing cyanobacteria in the Baltic Sea during the summer. The increase in the cyanobacterial population then leads to a further decrease of available P sources triggering by so far unknown mechanisms a subsequent mass development of filamentous N_2_-fixing cyanobacterial strains. These nutrient relations are the prevailing conditions in summer when the upper water layer is thermally stratified. The gas vesicles of *N. spumigena* and other bloom-forming cyanobacteria provide buoyancy, leading to the formation of large surface scums in the absence of mixing. A correlation between biologically available dissolved inorganic and organic P forms and *Nodularia* spp. bloom formation is also evident in the Baltic Sea (Nausch et al., 2008; Vahtera et al., 2010). Moreover, increased expression of the gene cluster for nodularin synthesis during P-depletion has been reported (Jonasson et al., 2008), although the amount of nodularin did not change at different P conditions (Repka et al., 2001).

Molecular regulation of the acclimation to different P availability has been intensively studied in model cyanobacteria such as *Synechocystis* sp. PCC 6803. Basically, the sensing and acclimation to P limitation seems to be similar to that in *Escherichia coli*, i.e., a two-component system PhoB/PhoR (SphB/SphR) senses the P status and induces a defined P-regulon under P-limiting conditions as has been shown using DNA-microarray-based transcriptomics (e.g., Suzuki et al., 2004). As in many other bacteria, the regulon comprises transporters for inorganic phosphate, such as two ABC type transporters of different affinity for orthophosphate (from here on *o*-phosphate) import, Pst1 and Pst2 (Pitt et al., 2010). Furthermore, alkaline phosphatase, an exoenzyme known to be involved in the release of *o*-phosphate from organic phosphates is part of the Sph-regulon in *Synechocystis* sp. PCC 6803 (Suzuki et al., 2004), which is typically overexpressed under P limitation in many other cyanobacteria as well (e.g., Kelly et al., 2019; Yuan et al., 2019). A similar role of PhoB and PhoR in the regulation of P-limitation associated genes has been shown for marine *Synechococcus* sp. WH8102 (Tetu et al., 2009). Subsequent studies revealed that an additional regulator protein PtrA is also necessary for the coordinated expression of P-regulated genes in this marine cyanobacterium (Ostrowski et al., 2010).

Furthermore, cyanobacteria and microalgae can accumulate polyphosphate that can serve as storage for excess phosphate and/or energy (Sanz-Luque et al., 2020). In enterobacteria as in model cyanobacteria such as *Synechocystis* sp. PCC 6803 polyphosphate accumulation is induced when cells are shifted from P-deplete into P-replete conditions to store the surplus phosphate inside the cell (Voronkov and Sinetova, 2019). Consistently, a knock-out mutant in polyphosphate degradation cannot properly acclimate to P limitation (Hiyoshi et al., 2021). However, there are also hints that polyphosphate accumulation and mobilization is not always strictly related to the P status among different cyanobacteria (Li et al., 2019; Wan et al., 2019). It has been reported that polyphosphate is stored in filamentous strains under P-deplete conditions without its mobilization to sustain growth (e.g., Hagemann et al., 2019), which may serve as P storage for the next year’s generation. In contrast to model strains such as *Synechocystis* sp. PCC 6803, environmentally important filamentous strains such as *Nodularia* spp. seem to have a much greater capability to deal with different P availability. For example, in the genome of *N. spumigena* strain CCY9414 many genes for the acquisition of different inorganic and organic P-sources have been annotated (Voss et al., 2013). In addition to *o*-phosphate, transporters and associated enzymes for the utilization of phosphonates and phosphites are present in its genome. Recently, it has been shown that many bacteria including diverse cyanobacterial species in marine and brackish systems can produce and utilize phosphonates to sustain growth under different nutrient availability (e.g., Acker et al., 2022; Rabouille et al., 2022; Zhao et al., 2022).

The Baltic Sea isolate *N. spumigena* strain CCY9414 (from here on *Nodularia* CCY9414) represents an ecologically relevant model organism suitable to analyze the molecular response to P limitation and elucidate its role in the formation of recurring summer blooms. Therefore, we grew *Nodularia* CCY9414 in P-replete and P-deplete media and compared polyphosphate accumulation as well as gene expression changes to gain insights into the acquisition and utilization of P sources under conditions representative of bloom events. Our results showed that P-limited cultures of *Nodularia* CCY9414 accumulate more polyphosphate than P-replete filaments despite a harsh P starvation as indicated by the induction of many genes related to low P availability. In addition to genes for transporters involved in the uptake of inorganic and organic P sources, genes for bioactive compounds, gas vesicle formation, and sugar metabolism were strongly up-regulated under P limitation in *Nodularia* CCY9414.

## 2. Materials and methods

### 2.1. Strain and cultivation

*Nodularia spumigena* is a toxic, filamentous planktonic, heterocystous, gas-vacuolate cyanobacterium, which is representative of surface bloom-forming cyanobacteria in brackish waters. The strain *N. spumigena* CCY9414 was initially isolated from samples collected from surface water in the Bornholm Sea (Hayes and Barker, 1997; Stal et al., 2003; Voss et al., 2013). Before the experiment, *Nodularia* CCY9414 cells were pre-cultivated in P-replete medium for 10 days to induce exponential growth. The P-replete medium was composed as follows: 33% ASNIII and 67% BG11 medium mixture [as described by Hagemann et al. (2019)], with 0.02 g L^–1^ K_2_HPO_4_ in the modified ASNIII (Rippka et al., 1979) and 0.00078 mg L^–1^ K_2_HPO_4_ in BG11 medium modified with 20 mM TES buffer to pH 8 (Rippka et al., 1979). Omitting the inorganic N-source nitrate induced N_2_-fixing conditions. Furthermore, the final NaCl concentration of the growth medium was set to 10.3 g L^–1^, which is corresponding to the salt optimum of *Nodularia* CCY9414 (Möke et al., 2013). Sterile cell culture flasks (Greiner Bio-One GmbH, Frickenhausen, Germany) were used for both pre-cultivation and afterward for the experimental cultures. Each culture flask contained 10 mL of starting culture suspension and 90 mL of fresh medium and was manually mixed daily. Incubations were performed at a temperature of 19.5 – 20°C at a light/dark cycle of 16 h/8 h. During the light phase, cell suspensions were constantly exposed to 40 μmol photons m^–2^ s^–1^. Every 5 days the cells were transferred into fresh P-replete medium to prolong the exponential growth until the planned start of the experiment.

At the beginning of the experiment, all the pre-cultures were combined and mixed in a glass bottle for a total volume of 500 mL. Filaments were then separated from the medium by filtration through glass fiber filters (25 mm circle diameter; GE Healthcare, Chicago, IL, United States). Half of the biomass on the filters were washed off and inoculated into 1.2 L of fresh medium but without added inorganic phosphate (P-deplete), which was then divided into 12 culture flasks with 100 mL suspension each. These P-deplete cultures contained only traces of inorganic phosphate impurities from other chemicals in the medium and from the inoculation of the pre-experimental cultures. The final concentration of *o*-phosphate was less than 1 μM. The remaining half of the biomass was washed off and inoculated into 1.2 L of fresh P-replete medium equally divided in 12 culture flasks. Samples were taken by sacrificing three culture flasks of each P condition at the beginning of the experiment (d0), after 7 (d7) and 14 days (d14), and at the end of the experiment after 21 days (d21). The experiment was repeated two times independently.

### 2.2. Dry weight and polyphosphate extractions

For dry weight estimations, three 5 mL aliquots from each culture were filtered on pre-weighted glass fiber filters of 25 mm diameter (GE Healthcare, Chicago, IL, United States) and dried at 65°C overnight. The mean dry weight of these three filters was taken to obtain a representative estimate for each culture flask.

Cells for polyphosphate quantification were collected from 3 mL aliquots on polycarbonate filters of 0.22 μm pore size and 22.5 mm diameter (GE Healthcare, Chicago, IL, United States), placed on ice and stored at −80°C until further processing. Extraction and fluorometric quantification of polyphosphate was done as described by Martin and Van Mooy (2013). Briefly, reagents were obtained from the following providers: Proteinase K (BP1700) was obtained from Sigma-Aldrich Chemie GmbH (Schnelldorf, Germany); lysozyme (BP535), ADP (A2754), Ambion recombinant DNase (AM2235) and RNase cocktail (AM2286), and DAPI (4′,6-diamidino-2-phenylindole) were from Thermo Fischer Scientific (Langenselbold, Germany). Before the polyphosphate extraction, cells on filter were dried for 5 h at 35°C to estimate their dry weight to standardize polyphosphate content. Then, cells were scraped off the filters with a spatula and resuspended in 3 mL polyphosphate buffer (20 mM HEPES, 100 mM NaCl, 2 mM EDTA, and 2 mM MgCl_2_). The dissolved polyphosphate extraction required eight freeze-thaw cycles (freeze at −20°C for 2 h and 30 min, thaw at 35°C for 40 min) for lysis of the cells. Subsequent boiling step and enzymatic digestion were performed as described in the method of Martin and Van Mooy (2013). To stain the dissolved polyphosphates 5 μL of DAPI (1 mg mL^–1^) were added to each extract. The measurements were made on a Tecan Infinite 200 PRO multimode plate reader (Tecan Austria GmbH, Grödig, Austria) at an excitation wavelength of 415 nm and emission wavelength of 550 nm, with an integration time of 500 μs. Fluorescence emission spectra were acquired at 415 nm and emission from 230 nm to 850 nm in 1-nm increments and integrated for 20 μs. All bandwidths were 5 nm. The calibration curve was constructed from commercial polyphosphate with a chain length of 45 ± 5 residues (Sigma-Aldrich, S4379) in the range of 0.2–7.0 nmol polyphosphates.

### 2.3. Inorganic phosphate content

During the second iteration of the experiment, aliquots of 10 mL were collected from each culture flask to measure the level of the *o*-phosphate (PO_4_^3–^) in the medium. Each aliquot was filtered through a glass fiber filter (25 mm circle diameter; GE Healthcare, Chicago, IL, United States), and *o*-phosphate and ammonium concentrations of the filtrate were measured colorimetrically according to Grasshoff et al. (2009) by means of a Seal Analytical QuAAtro constant flow analyzer (Seal Analytical GmbH, Norderstedt, Germany).

### 2.4. DAPI staining and epifluorescence microscopy

An aliquot of 1.5 mL was collected from each culture, fixed with 4% formaldehyde (v/v) and stored at −20°C until DAPI staining. After thawing, the filaments were harvested on polycarbonate membrane filters (0.88 μm pore size, 22.5 mm circle diameter, GE Healthcare, Chicago, IL, United States) and stained with 50 μl of DAPI solution (1 mg mL^–1^, Thermo Fischer Scientific, Langenselbold, Germany). Staining was performed in the dark at room temperature for 2 min. Then, the dye was filtered off, the filter was rinsed with ultra-pure water and dried in the dark at room temperature for 5 min. Polyphosphates in the stained filaments were visualized under a fluorescence microscope (Axioskop 2 mot PLUS, Carl Zeiss, Jena, Germany) with the specific DAPI filter set (excitation: BP 390/22, beam Splitter: FT 420, emission: 460/50). Polyphosphate chains longer than 15 P-subunits formed a complex with the dye that increased its fluorescence and shifted its absorption spectrum from 456 nm – when only DNA is visualized via blue fluorescence emission – to 526 nm via bright yellow fluorescence of the accumulated polyphosphate granules (Tijssen et al., 1985; Ohtomo et al., 2008; Diaz and Ingall, 2010).

### 2.5. RNA isolation

Filaments from aliquots of 30 mL of each culture were transferred to a 50 mL tube (Sarstedt, Nümbrecht, Germany) and immediately fixed for RNA extraction with 6 mL of a solution containing 95% (v/v) ethanol (molecular biology grade; Roth, Karlsruhe, Germany) and 5% (v/v) Roti-Aqua-Phenol (Roth, Karlsruhe, Germany). The fixed suspension was incubated in the dark at room temperature for 20 min to complete the collapse of gas vesicles before each sample was placed on ice. Then, cells were harvested by centrifugation at 12.000 *g* at 4°C for 8 min. The supernatant was quickly removed, and the collected pellets were flash frozen in liquid nitrogen until being processed for RNA isolation. The cell pellets were then suspended in 1 mL TRIzol reagent (Sigma-Aldrich, Steinheim am Albuch, Germany) and three spoons of acid-washed mixed glass beads of 425–600 μm (Sigma-Aldrich, Steinheim am Albuch, Germany) were added for bead beating for 3 × 30 s. Then, the suspension was heated at 65°C for 10 min and RNA was isolated as described by Steunou et al. (2006). RNA extracts were treated three times with two units of DNase I RNase-free (New England Biolabs, Frankfurt am Main, Germany) for 30 min as recommended by the manufacturer. DNase I was inactivated and removed with phenol/chloroform and total RNA was then precipitated with 3 M Na-Acetate pH 5.2 and 2.5 volumes of absolute Ethanol. RNA extracts were checked for DNA contamination by PCR using primers specific for the gene encoding Fe-superoxide dismutase subunit B (*sodB* forward: GACTCCTCTAAGGTGGGAATC; *sodB* reverse: CCCAGACATCCAAGGTTAAG) as was previously done by Kopf et al. (2015).

### 2.6. cDNA library preparation and sequence processing

Non-stranded rRNA-depleted libraries were prepared by the sequencing company LGC (LGC, Biosearch Technologies, Berlin, Germany) for 12 samples, collected at d7 and d14 for both P conditions from the second iteration of the experiment. Briefly, the total RNA was first depleted of rRNA using the Pan-Prokaryote riboPOOL kit (siTOOLs Biotech). The RNA was then converted into cDNA using the NEBNext RNA First Strand Synthesis and NEBNext RNA Second Strand Synthesis Modules (New England Biolabs). For the preparation of the indexed Illumina libraries the Encore Rapid DR Multiplex System 1–96 (NuGEN) was used. Libraries were amplified with 12 cycles and sequenced on Illumina NextSeq500/550.

Between 6.8 and 11.6 million single 75 bp reads were generated per sample. Adapter-clipped reads provided by the sequencing company were quality trimmed with BBDuk (Bushnell, 2014) using a sliding window approach with a window size of 4 bp and an average base quality of 15. Poly-G repeats longer than 10 bp were removed and reads shorter than 50 bp were discarded. Quality-trimmed reads were mapped against the reference genome of *Nodularia* CCY9414 (NCBI RefSeq accession: GCF_000340565.2) using the program bwa-mem (Li, 2013). Remaining hits to ribosomal RNA genes were excluded. Mapping results were further filtered to remove secondary and supplementary alignments, as well as alignments shorter than 50 bp and those with less than 95% sequence identity across the whole read to the reference. Read counts per gene were then calculated with featureCounts (Love et al., 2014) and converted to transcript percentages accounting for variable gene length. Operons were predicted with OperonMapper^1^ (Taboada et al., 2018). To perform a functional enrichment analysis based on the KEGG pathway hierarchy, the reference genome of *Nodularia* CCY9414 was re-annotated against KEGG (release January 2022) using diamond blastp version 2.0.14.152 (Buchfink et al., 2021) in sensitive mode, retaining hits with an e-value below 1e-5 and a blast score ratio of more than 0.4. KEGG ortholog (KO) annotations were assigned based on the best hit. Genes without a KO assignment in the blastp search were attributed a KO number according to kofamscan version 1.3.0 (Aramaki et al., 2019) at an *e*-value of 0.01. Mapping of genes to KEGG pathways was performed based on KO assignments, excluding pathways from overview and structural maps and those exclusive to viruses or eukaryotic organisms. The transcriptomic reads and processed feature counts are accessible on the GEO database^2^ with the following accession number: GSE213384.

### 2.7. Statistical data analysis

All the statistical data analyses and visualization were performed in R (R Core Team, 2021) using the additional packages tidyverse (Wickham et al., 2019), DESeq2 (Love et al., 2014), vegan (Oksanen et al., 2020), car (Fox and Weisberg, 2019), emmeans (Lenth, 2022), multcomp (Hothorn et al., 2008), multcompView (Graves et al., 2019), lmerTest (Kuznetsova et al., 2017), hrbrthemes (Rudis, 2020), and RColorBrewer (Neuwirth, 2014). Post-processing of the figures was performed using the software Inkscape (Inkscape Project, 2020). Further details about the bioinformatic sequence processing and statistical data analysis are available on http://doi.io-warnemuende.de/10.12754/misc-2022-0005.

Dry weight and polyphosphate concentrations were analyzed in a generalized linear mixed model to assess the effect of sampling time point and P conditions with experiment iteration as random factor. To meet the assumption of normality, polyphosphate concentrations were square-root transformed. Outlier observations with a Cook’s distance of more than 4 divided by sample size (Cook and Weisberg, 1984; Williams, 1987) were removed from the analysis. *Post hoc* tests, i.e., pairwise comparisons between experimental conditions and sampling time points, were implemented in emmeans (Lenth, 2022). Differential gene expression was assessed using DESeq2 (Love et al., 2014) between P-replete and P-deplete conditions at each sampling time point and between d7 and d14 in each P treatment. Genes were detected as differentially expressed at a Benjamini–Hochberg adjusted p-value of 0.1 and an absolute log2-fold change of at least 1. Functional enrichment analysis was conducted for each pathway using the proportion of genes per pathway of the total number of genes in the genome in a X^2^ goodness-of-fit analysis. The aim was to assess if the number of differentially expressed genes per pathway was higher than expected by chance. Significance was assessed at a family-wise error rate of 0.05 after Benjamini–Hochberg adjustment of *p*-values to account for multiple testing. Cases with expected frequencies below 1 were marked as potentially unreliable in the results.

## 3. Results

### 3.1. Physiological and biochemical characterization of P-limitation

**FIGURE 1:**
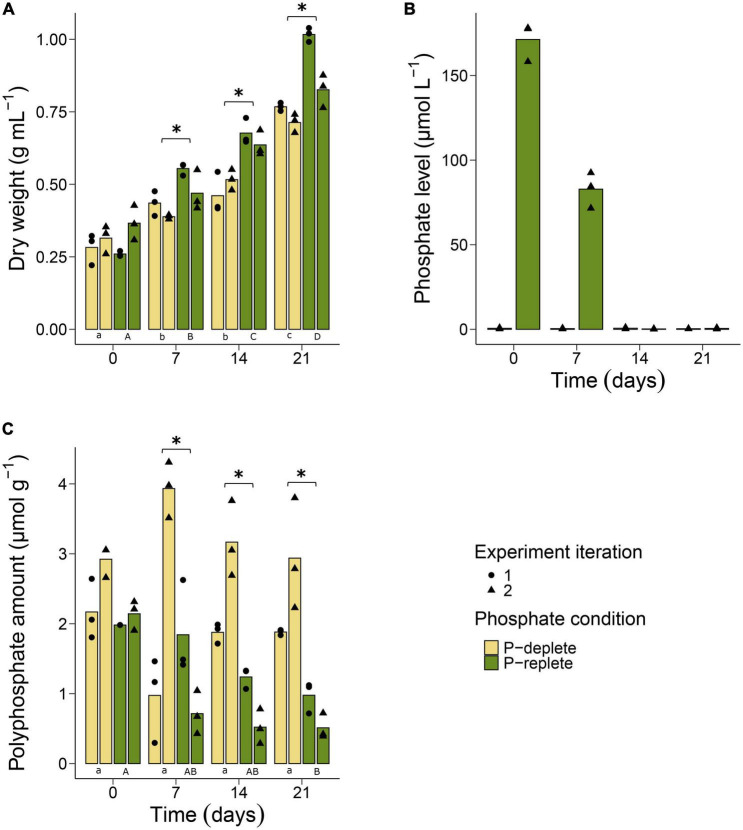
Physiological and biochemical assessment of *Nodularia spumigena* CCY9414 under P-replete (+P) and P-deplete (–P) conditions. Growth expressed as increase in dry weight **(A)**. *O*-phosphate (PO_4_^3–^) concentration in the cultivation media during the second experiment iteration **(B)**. Accumulation of polyphosphates in cells of *N. spumigena* **(C)**. Bars represent means of individual measurements shown as single points. Asterisks indicate significant differences between the P-replete and the P-deplete cultures at each sampling time point. Significant differences between sampling time points are indicated by different lower-case (P-deplete) and upper-case (P-replete) letters. The results of the statistical tests can be found in the Supplementary Tables 1, 2.

All experiments were performed under conditions that are prevailing in the Baltic Sea during *Nodularia* spp. summer blooms. Dry weight (DW) measurements were performed for each culture in both conditions (P-deplete and P-replete) to evaluate growth throughout the two independent experiments. The increase in biomass was faster in P-replete than in P-deplete conditions (Figure 1A). However, even the cyanobacteria in the P-deplete medium were able to almost triple their biomass throughout the 3 weeks’ incubation time, whereas P-replete conditions permitted a fourfold increase.

In addition, *o*-phosphate and ammonium concentrations were measured weekly. In media of P-deplete cultures, the amount of *o*-phosphate was always near the detection limit with values between 0.9 and 0.2 μM (Figure 1B). In the P-replete medium, *o*-phosphate concentrations were approximately 170 μM and dropped during the first 7 days of the experiment to about 50% of the initial amounts. Almost all *o*-phosphate was consumed by the cells in the P-replete conditions after 14 days, i.e., at that time point similarly low *o*-phosphate levels were detected in P-deplete and P-replete cultures. In contrast to *o*-phosphate, similar levels of ammonium, released from the N_2_-fixing filaments, were found in P-replete and P-deplete cultures during the entire experimental period. Its amount was always approximately 100 μM and did not differ significantly with time between the two experimental treatments (Supplementary Figure 1).

Polyphosphates were quantified in filaments at all sampling time points in P-replete and P-deplete cultures in two independent experiments (Figure 1C). At time point 0, cultures of both P conditions started with a relatively high internal polyphosphate amount of approximately 2.5 μmol g^–1^ DW. This amount continuously decreased in filaments grown under P-replete conditions, although the rate of decrease differed between the two experiment iterations. Especially in the second iteration of the experiment, the polyphosphate content decreased to less than 50% already after 7 days, when still substantial amounts of *o*-phosphate were available for the cells (Figure 1B). In contrast, filaments grown under P-deplete conditions did not significantly alter their internal polyphosphate pool during the 3-week time period, despite the observed divergent measurements between the experiment iterations at day 7 (Figure 1C). In addition to the chemical quantification of polyphosphate, we used DAPI-staining to obtain a qualitative picture of polyphosphate accumulation in different cell types of the *Nodularia* CCY9414 filaments. Stained filaments from P-replete and P-deplete cultures showed yellow inclusions at each time point in vegetative cells and in heterocysts (Supplementary Figure 2).

### 3.2. RNA-seq characterization of P-limitation

RNA was isolated and sequenced from *Nodularia* CCY9414 cells cultivated for 7 and 14 days (d7 and d14) under P-replete and P-deplete conditions, respectively, during the second experiment iteration. The RNA-seq approach enabled the differential expression analysis of 4752 non-ribosomal genes (Supplementary Data 1). Thereby, a larger number of genes showed increased compared to decreased expression in the three comparisons: day 7 P-replete relative to day 7 P-deplete conditions, day 14 P-replete relative to day 14 P-deplete conditions, and day 14 P-replete relative to day 7 P-replete conditions.

**FIGURE 2:**
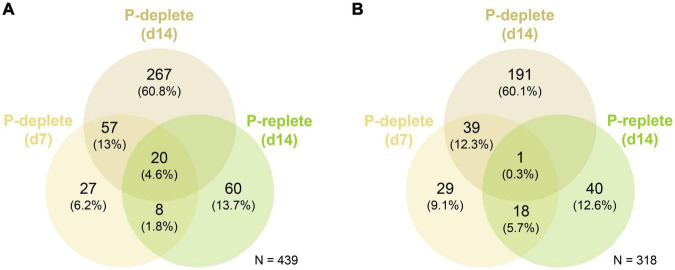
Venn diagrams for the global quantitative comparison of expression changes in *Nodularia spumigena* CCY9414 under P-replete (+P) or P-deplete (–P) conditions. Number of up-regulated **(A)** and down-regulated **(B)** genes under P starvation in the comparison of P-deplete and P-replete conditions at day 7, P-deplete and P-replete conditions at day 14, and between P-replete conditions at day 14 and day 7, respectively. The RNA-seq experiment was conducted with samples from the second experiment iteration.

At day 7, 112 genes were up-regulated and 87 down-regulated comparing P-deplete and P-replete cultures, respectively, whereas these numbers increased after 14 days to 344 up-regulated and 231 down-regulated genes (Figure 2). Among them, 77 genes were commonly up-regulated in P-deplete cultures at the two time points and 40 remained down-regulated. Furthermore, the gene expression changes at day 14 compared to day 7 in P-replete cultures resembled the changes observed in P-deplete versus P-replete conditions when compared at the same sampling time point. Overall, 28 of the 88 up-regulated genes at day 14 in P-replete cultures were also up-regulated at day 7 in P-deplete *Nodularia* CCY9414 filaments (Figure 2). A similar relative overlap is also found between down-regulated genes in day 14 P-replete relative to day 7 P-deplete (each compared to day 7 P-replete) cultures. These relations indicate that during the long-term growth under initially P-replete conditions many P-limitation-induced genes became up-regulated, which is consistent with the complete consumption of *o*-phosphate between 7 and 14 days in these cultures (Figure 1B).

**FIGURE 3:**
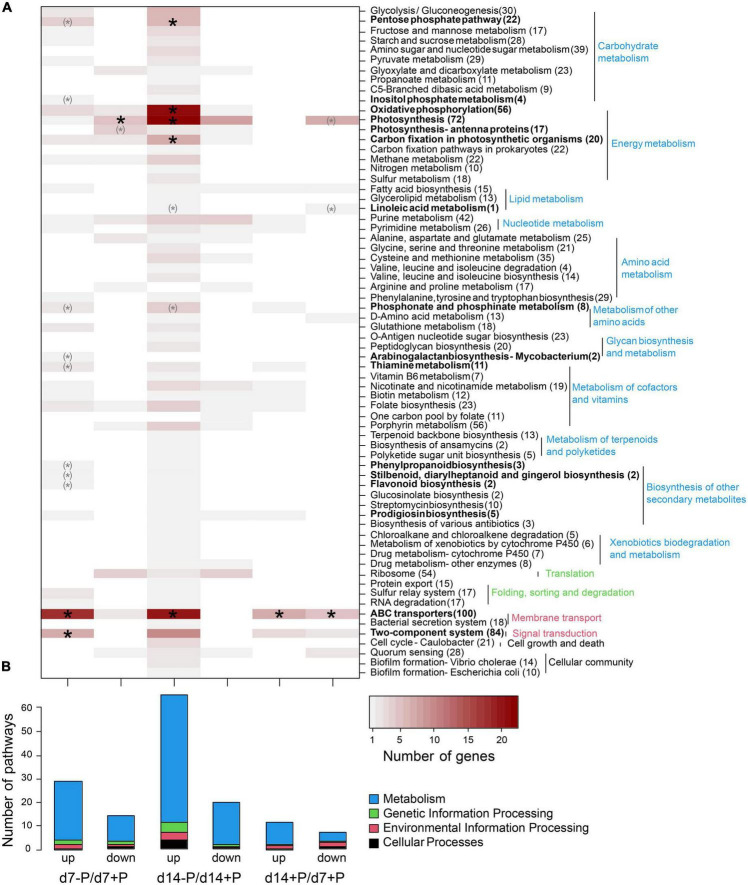
Assignment of differentially expressed genes to KEGG pathways. Up- and down-regulated genes are shown for the comparison of P-deplete and P-replete conditions at d7, P-deplete and P-replete conditions at d14, and between P-replete conditions at d14 and d7. **(A)** Number of differentially expressed genes per KEGG pathway. Asterisks indicate significant functional enrichment (X^2^ goodness-of-fit test, *p* < 0.05, parentheses indicate expected frequencies below 1). Number of genes per pathway in the genome is given after the pathway name. **(B)** Number of pathways containing differentially expressed genes. The RNA-seq experiment was conducted with samples from the second experiment iteration.

Specific functional pathways were over-represented among the differentially expressed genes under specific P conditions (Figure 3). Especially at day 7, a disproportionally large number of genes up-regulated under P-deplete conditions were recruited from ABC transporters, among them 17 transporters for different P sources, and many genes for phosphonate and phosphinate metabolism. In addition, there were hints that P starvation had marked influence on the overall cell metabolism, e.g., many genes encoding regulatory proteins from two-component systems or enzymes involved in the metabolism of secondary metabolites as well as the oxidative pentose-phosphate (OPP) pathway were higher expressed, whereas genes for photosynthetic complexes or enzymes of the Calvin-Benson cycle appeared down-regulated at day 7 (Supplementary Data 1). After 14 days of growth without P, many more genes became stimulated. In addition to the previously mentioned P-associated genes, genes for proteins involved in energy metabolism, such as photosynthesis, oxidative phosphorylation and carbon fixation, were among the functional groups enriched in low P-stimulated genes after 14 days (Figure 2). Many genes encoding P limitation-related transport or regulatory proteins also became higher expressed when we compared the gene expression of P-replete cultures at day 14 to day 7, when all available *o*-phosphate had been consumed from the medium (Figure 1B).

### 3.3. Defining the P-specific stimulon

In the initial genome annotation study, the authors provided a comprehensive overview of putatively P-associated genes in *Nodularia* CCY9414 (Voss et al., 2013). To confirm their involvement in the acclimation to P-limitation in our experimental set-up, the relative expression of all these genes was reviewed in the RNA-seq data (Supplementary Table 3 and Supplementary Data 1). This comparison permitted an experimentally supported annotation of the specific P-related stimulon in *Nodularia* CCY9414 (Table 1).

**TABLE 1:** P-related genes in *Nodularia spumigena* CCY9414 according to Voss et al. (2013) and their differential expression after 7 or 14 days of P-limitation as well as during growth for 14 days in P-replete medium.

Old locus tag	New locus tag	Product	d7-P/d7+P	d14-P/d14+P	d14+P/d7+P	Operon
**Inorganic P transport**						
nsp28900	NSP_RS12745	Periplasmic P binding protein PstS (similar to slr1247, high affinity, low velocity Pst2 system in PCC6803)	**7.08 ± 0.13**	**4.33 ± 0.14**	**3.26 ± 0.16**	1909
nsp28910	NSP_RS12750	PstC component of high affinity ABC P transporter	**5.05 ± 0.17**	**3.36 ± 0.15**	**1.87 ± 0.21**	1909
nsp28920	NSP_RS12755	PstA component of high affinity ABC P transporter	**4.16 ± 0.25**	**3.22 ± 0.22**	**1.31 ± 0.34**	1909
nsp28930	NSP_RS12760	PstB component of high affinity ABC P transporter ATP-binding protein component	**4.03 ± 0.26**	**3.88 ± 0.20**	*0.89* ± *0.31*	1909
nsp52600	NSP_RS23150	Periplasmic P binding protein PstS (similar to sll0680, low affinity, high velocity Pst1 system in PCC6803)	**3.72 ± 0.14**	**1.85 ± 0.12**	**2.24 ± 0.14**	3425
nsp52610	NSP_RS23155	PstC component of high affinity ABC P transporter	**2.85 ± 0.13**	**2.16 ± 0.12**	**1.54 ± 0.12**	3425
nsp52620	NSP_RS23160	PstA component of high affinity ABC P transporter	**2.30 ± 0.18**	**2.32 ± 0.12**	*0.96* ± *0.13*	3425
nsp52630	NSP_RS23165	PstB component of high affinity ABC P transporter ATP-binding protein component	**1.93 ± 0.12**	**1.50 ± 0.10**	*0.75* ± *0.12*	3425
**Phosphonate transport**						
**nsp7590**	NSP_RS03415	PhnF component of a C-P lyase	*−0.35* ± *0.18*	*−0.32* ± *0.20*	*−0.43* ± *0.19*	516
**nsp7580**	NSP_RS03410	PhnG component of a C-P lyase	**1.54 ± 0.23**	**1.38 ± 0.22**	*0.66* ± *0.27*	515
**nsp7570**	NSP_RS03405	PhnH component of a C-P lyase	**2.15 ± 0.31**	**1.96 ± 0.25**	**1.01 ± 0.38**	515
**nsp7560**	NSP_RS03400	PhnI component of a C-P lyase	*0.90* ± *0.17*	*0.98* ± *0.14*	*0.41* ± *0.18*	515
**nsp7550**	NSP_RS23855	VOC family protein (similar to PhnM of *Nostoc sphaeroides*)	*2.86* ± *0.89*	*1.01* ± *0.59*	*2.24* ± *1.00*	515
**nsp7540**	NSP_RS03395	PhnJ component of a C-P lyase	*0.92* ± *0.29*	*0.94* ± *0.26*	*0.45* ± *0.32*	515
**nsp7530**	NSP_RS03390	PhnK component of a C-P lyase	**1.22 ± 0.24**	*0.79* ± *0.23*	*0.98* ± *0.28*	515
**nsp7520**	NSP_RS03385	PhnL component of a C-P lyase	*0.59* ± *0.34*	**1.10 ± 0.30**	*0.24* ± *0.37*	515
**nsp7510**	NSP_RS03380	PhnM component of a C-P lyase	*0.82* ± *0.25*	**1.27 ± 0.22**	*0.32* ± *0.29*	515
**nsp7500**	NSP_RS03375	Hypothetical protein in phn cluster	*0.99* ± *0.32*	*0.49* ± *0.29*	*0.52* ± *0.37*	515
**nsp7490**	NSP_RS03370	Hypothetical protein in phn cluster	*1.30* ± *0.48*	*0.97* ± *0.51*	*0.31* ± *0.63*	514
**nsp7480**	NSP_RS03365	PhnD component of phosphonate ABC transporter phosphate-binding periplasmic component	**7.23 ± 0.32**	**5.81 ± 0.18**	**2.82 ± 0.33**	513
**nsp7470**	NSP_RS03360	PhnC phosphonate ABC transporter ATP-binding protein	**4.69 ± 0.25**	**5.00 ± 0.19**	*0.91* ± *0.29*	513
**nsp7460**	NSP_RS03355	PhnE phosphonate ABC transporter permease	**4.36 ± 0.25**	**4.41 ± 0.20**	*0.97* ± *0.29*	513
**nsp7450**	NSP_RS03350	PhnE3 phosphonate ABC transporter permease	**3.64 ± 0.20**	**3.87 ± 0.16**	*0.70* ± *0.23*	513
nsp35120	NSP_RS15575	PhnC1 phosphonate ABC transporter ATP-binding protein	**3.54 ± 0.20**	**2.67 ± 0.17**	*0.83* ± *0.27*	2314
nsp35130	NSP_RS15580	PhnD1 phosphonate ABC transporter phosphate-binding periplasmic component	**1.05 ± 0.15**	*0.91* ± *0.11*	*0.26* ± *0.14*	2314
nsp35140	NSP_RS15585	PhnE1 phosphonate ABC transporter permease protein	**1.20 ± 0.15**	**1.31 ± 0.16**	*0.00* ± *0.18*	2314
nsp35150	NSP_RS15590	PhnH (truncated version, translationally coupled to nsp35160 – phnM component of a C-P lyase)	*1.43* ± *0.45*	**1.12 ± 0.49**	*0.14* ± *0.59*	2314
**nsp18360**	NSP_RS08220	PhnD2 phosphonate ABC transporter phosphate-binding periplasmic component	**−1.53 ± 0.14**	*0.43* ± *0.17*	**−1.42 ± 0.13**	1262
**nsp18370**	NSP_RS08225	PhnC2 phosphonate ABC transporter ATP-binding protein	**−***0.96* ± *0.16*	*0.26* ± *0.16*	**−***0.94* ± *0.16*	1262
**nsp18380**	NSP_RS08230	PhnE2 phosphonate ABC transporter permease protein	**−***0.53* ± *0.20*	*0.37* ± *0.18*	**−***0.72* ± *0.20*	1262
**Phosphite transport**						
nsp35050	NSP_RS15540	PtxA phosphite ABC transporter permease protein	**3.29 ± 0.21**	**2.78 ± 0.17**	**1.24 ± 0.24**	2311
nsp35060	NSP_RS15545	PtxB phosphite ABC transporter phosphate-binding periplasmic component	**2.39 ± 0.20**	**2.76 ± 0.18**	*0.68* ± *0.22*	2311
nsp35070	NSP_RS15550	PtxC phosphite ABC transporter permease protein	**1.60 ± 0.15**	**1.90 ± 0.13**	*0.40* ± *0.17*	2311
nsp35080	NSP_RS15555	Phosphite dehydrogenase, 2-hydroxyacid dehydrogenase	**1.23 ± 0.14**	**1.68 ± 0.13**	*0.15* ± *0.16*	2311
nsp35090	NSP_RS15560	LysR transcriptional regulator	**1.69 ± 0.30**	**1.20 ± 0.26**	*0.88* ± *0.35*	2312
**P storage and degradation of P polymers**						
nsp10230	NSP_RS04600	Ppk polyphosphate kinase (ppk)	*0.48* ± *0.14*	**1.21 ± 0.12**	**−***0.11* ± *0.13*	706
**Degradation of organic P sources**						
nsp6490	NSP_RS02895	Glycerophosphoryl diester phosphodiesterase (phytase domain)	**4.63 ± 0.18**	**5.45 ± 0.15**	*0.90* ± *0.13*	448
nsp7010	NSP_RS03145	Atypical alkaline phosphatase (esterase-like activity of phytase family protein)	**5.53 ± 0.22**	**3.82 ± 0.12**	**3.17 ± 0.13**	481
nsp7000	NSP_RS03140	Metallophosphoesterase	**4.38 ± 0.20**	**3.42 ± 0.14**	**2.18 ± 0.17**	480
nsp6990	NSP_RS03135	DUF4114 domain-containing protein	**2.55 ± 0.14**	**2.47 ± 0.12**	*0.90* ± *0.14*	480
nsp12920	NSP_RS05785	Alkaline phosphatase, extracellular	**6.64 ± 0.23**	**7.75 ± 0.41**	**1.07 ± 0.21**	904
nsp12930	NSP_RS05790	Cation diffusion facilitator family transporter	**7.14 ± 0.16**	**4.97 ± 0.32**	**2.74 ± 0.23**	904
nsp12940	NSP_RS05800	PhoX-like phosphatase	**7.02 ± 0.17**	**5.41 ± 0.14**	**2.84 ± 0.14**	905
nsp18960	NSP_RS08485	Putative PhoX phosphatase, DUF839 domain-containing protein	**6.28 ± 0.15**	**5.69 ± 0.15**	**1.36 ± 0.17**	1304
nsp29340	NSP_RS12935	Metallophosphoesterase	**−1.82 ± 0.41**	**−***0.67* ± *0.10*	**−1.16 ± 0.37**	1934
nsp29350	NSP_RS12940	Metallophosphoesterase	**−***0.10* ± *0.15*	*0.00* ± *0.12*	**−***0.06* ± *0.15*	1934
nsp53310	NSP_RS23475	Alkaline phosphatase D family protein	**5.39 ± 0.13**	**5.42 ± 0.14**	*0.91* ± *0.15*	3474
**Arsenate-related gene orthologs/operons**						
nsp33490	NSP_RS14830	ArsR (regulator of arsenate resistance)	**−1.21 ± 0.15**	**−1.14 ± 0.17**	**−***0.78* ± *0.18*	2213
nsp33500	NSP_RS14835	SphX periplasmic P binding component of P ABC transporter	*0.75* ± *0.14*	**1.21 ± 0.13**	**−***0.22* ± *0.15*	2213
nsp33510	NSP_RS14840	ArsJ associated glyceraldehyde-3-phosphate DH	**1.57 ± 0.26**	**2.41 ± 0.23**	*0.09* ± *0.29*	2214
nsp33520	NSP_RS14845	ArsJ, major facilitator superfamily permease	*0.29* ± *0.20*	**1.20 ± 0.14**	**−***0.25* ± *0.17*	2215

**FIGURE 4:**
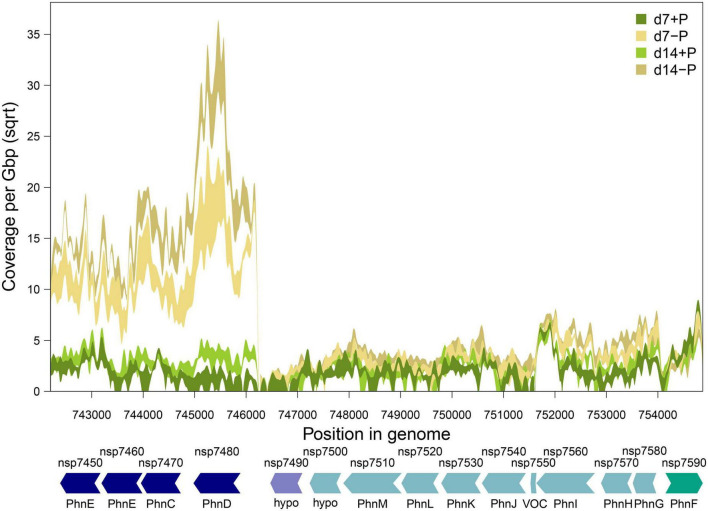
Gene expression of phosphate-regulated gene cluster *nsp7450* to *nsp7590* in *N. spumigena* CCY9414 under P-replete (+P) and P-deplete (–P) conditions. Shaded bands show minimum and maximum gene expression (normalized as square-root transformed per base pair coverage per Gbp sequencing effort) at each experimental condition. Arrows indicate gene arrangement and orientation and were colored according to operon assignment as provided in Table 1. Locus tags and gene names are according to Table 1 (hypo: hypothetical protein).

Significantly differentially expressed genes were identified at an absolute log_2_-fold change ≥ 1 and a Benjamini–Hochberg adjusted *p*-value of ≤0.1. Log_2_-fold changes are given with standard error (*n* = 3) in bold (significant) or italics (non-significant change). Bold locus tag names form a very large P-regulated cluster on the *Nodularia* CCY9414 chromosome (Figure 4). Only genes of the annotated P stimulon according to Voss et al. (2013) exhibiting strong responses to P-starvation are shown. The full list of differentially expressed genes is provided in Supplementary Data 1.

Approximately half of the previously suggested P-associated genes (Voss et al., 2013) were among the differentially expressed genes identified in our experiment, comprising genes which exhibited the strongest responses including transport systems for different inorganic and organic P-sources (Supplementary Figure 3). Among them were two Pst systems (organized in operons 1909 and 3425) for *o*-phosphate that were concordantly stimulated but to a different extent. The operon 1909 that encodes for proteins similar to the high affinity but low velocity Pst2 system in *Synechocystis* sp. PCC 6803 (Pitt et al., 2010) is about three times higher expressed than the genes for the lower affinity but higher velocity Pst1 system. In addition, a large chromosomal region was found on which many P-regulated genes were situated. It comprises the genes *nsp7450* to *nsp7590* that encode at least two phosphonate transport systems in the operons 515 and 513, which were induced after 7 as well as 14 days of P-limitation (Table 1 and Figure 4). Interestingly, a third annotated phosphonate uptake system encoded by the operon 1262 was weakly down-regulated after 7-day growth under P-deplete conditions (Table 1). Moreover, the phosphite ABC transporter encoded by the operon 2311 was also stimulated after 7 and 14 days of P-limitation. In addition, many genes encoding alkaline or acid phosphatases, phytases and related proteins known to be able to release *o*-phosphate from different organic P-sources belonged to the most strongly induced genes. Among them, the alkaline phosphatase NSP_RS05785 was predicted with high confidence by TargetP^3^ to possess a transit peptide at the N-terminus, which indicates that this enzyme is likely released from the cell to acquire extracellular P (Table 1).

However, many genes that were previously suggested to be part of the P-stimulon (Voss et al., 2013) did not show pronounced phosphate-related gene expression changes, such as metallophosphoesterases, haloacid dehalogenase-like hydrolases and the majority of the arsenate-related genes with a few exceptions (Supplementary Table 3 and Supplementary Data 1). Furthermore, most genes annotated to be involved in polyphosphate synthesis and mobilization did not clearly change the expression upon P-limitation. Only the *ppk* gene, encoding the polyphosphate synthesizing kinase, became significantly higher expressed after 14 days of growth under P-deplete conditions, which was consistent with the largely stably maintained polyphosphate pool in these filaments (Figure 1C). Finally, despite the induction of many transporters for different P-sources, the expression of the related transcriptional regulators, i.e., the two-component regulatory system PhoB (SphR) and PhoR (SphS), were not significantly changed (Supplementary Table 3 and Supplementary Data 1). Only one LysR-type transcriptional regulator that is found downstream of the operon 2311 encoding the phosphite-specific ABC transporter showed significantly enhanced expression under P-limiting conditions (Table 1).

### 3.4. Further P-regulated genes

In addition to the above-mentioned genes encoding proteins that were likely directly involved in the acclimation to P limitation, many more genes appeared to be directly or indirectly P-regulated in *Nodularia* CCY9414. Amongst them, many genes encode hypothetical proteins with no functional annotation, i.e., at the time point day 7 this group comprised approximately 25% all up-regulated genes under P-deplete conditions (Supplementary Data 1). However, several functionally annotated and physiologically important genes were also strongly induced in P-starved cells (Table 2). One example is represented by three genes (new locus tags NSP_RS14115, NSP_RS14120, and NSP_RS14125) encoding proteins involved in the synthesis of a CTB family bacteriocin, which were induced after 7 and 14 days of growth in the P-deplete medium. The genome of *Nodularia* CCY9414 harbored a second cluster of genes (NSP_RS06270, NSP_RS06275, NSP_RS06280, NSP_RS06285, and NSP_RS06290) for bacteriocins, which was not differentially expressed under different P conditions (Supplementary Data 1). Furthermore, several genes associated with bloom formation of *Nodularia* CCY9414 appeared to be up-regulated under P starvation. Amongst them, several of the encoded proteins were related to N_2_ fixation, such as the Mo-dependent nitrogenase C-terminal domain-containing protein (NSP_RS06835), the nitrogen fixation protein NifX (NSP_RS18010), the nitrogenase-stabilizing/protective protein NifW (NSP_RS18025), which were higher expressed at day 7 in N-deplete filaments (Table 2). Additional bloom-related genes became induced after 14 days of growth under P-deplete conditions. Notably, a cluster of genes encoding different gas vesicle constituents, such as GvpA and GvpC (operon 1062: NSP_RS06890, NSP_RS06895, NSP_RS06900, as well as NSP_RS06910), or a non-ribosomal peptide synthetase operon 3225 (NSP_RS21715, NSP_RS21720, and NSP_RS21725) were among them. It further seemed that after 2 weeks of P starvation, the iron homeostasis in *Nodularia* CCY9414 was affected, because genes for iron uptake systems, such as the TonB receptor and the iron-siderophore ABC transporter substrate-binding protein (NSP_RS05344 and NSP_RS05350), as well as iron ABC transporter permeases (NSP_RS05360 and NSP_RS05365) became up-regulated (Table 2). Finally, the sugar-phosphate metabolism appeared to be altered due to strong P-limitation. Especially genes for enzymes involved in the OPP pathway, including the entrance enzyme glucose 6-phosphate dehydrogenase (*zwf*, NSP_RS22185), were found to be up-regulated after 7 and 14 days of P starvation.

**TABLE 2:** Expression changes of genes encoding proteins indirectly involved in P acclimation of *N. spumigena* CCY9414 after 7 or 14 days of P-limitation as well as during growth for 14 days in P-replete medium.

Old locus tag	New locus tag	Product	d7-P/d7+P	d14-P/d14+P	d14+P/d7+P	Operon
**Iron/metal homeostasis**						
nsp11930	NSP_RS05350	Iron-siderophore ABC transporter substrate-binding protein	*−0.14* ± *0.41*	**1.08 ± 0.45**	*−0.64* ± *0.49*	830
nsp11940	NSP_RS05360	Iron ABC transporter permease	*−0.14* ± *0.26*	**2.64 ± 0.26**	−**1.27 ± 0.30**	831
nsp11950	NSP_RS05365	Iron ABC transporter permease	*−0.18* ± *0.15*	**1.63 ± 0.16**	−**1.22 ± 0.17**	831
nsp15240	NSP_RS06840	Cupin domain-containing protein	**1.18 ± 0.15**	**1.99 ± 0.15**	*−0.49* ± *0.17*	1054
**Gas vesicle**						
nsp15380	NSP_RS06890	Gas vesicle structural protein GvpA	*0.20* ± *0.40*	**2.14 ± 0.14**	−**1.11 ± 0.35**	1062
nsp15390	NSP_RS06895	Gas vesicle structural protein GvpA	*0.76* ± *0.17*	**2.02 ± 0.13**	*−0.39* ± *0.15*	1062
nsp15400	NSP_RS06900	Gas vesicle protein GvpC	*0.80* ± *0.13*	**1.61 ± 0.11**	*−0.16* ± *0.12*	1062
nsp15420	NSP_RS06910	Gas vesicle protein	*0.41* ± *0.11*	**1.13 ± 0.12**	*−0.15* ± *0.11*	1064
nsp35630	NSP_RS15765	Gas vesicle structural protein GvpA	*0.18* ± *0.40*	**2.14 ± 0.13**	−**1.14 ± 0.34**	2343
**Toxin/bioactive compound synthesis**						
nsp8480	NSP_RS03815	HlyD family efflux transporter periplasmic adaptor subunit	*0.43* ± *0.19*	**1.09 ± 0.14**	*−0.15* ± *0.17*	580
nsp26940	NSP_RS11895	Type I polyketide synthase	−**1.08 ± 0.13**	*0.78* ± *0.16*	−**2.05 ± 0.14**	1796
nsp31980	NSP_RS14125	CTB family bacteriocin	**1.51 ± 0.15**	**1.75 ± 0.16**	*0.21* ± *0.13*	2107
nsp31970	NSP_RS14120	CTB family bacteriocin	**1.10 ± 0.14**	**1.45 ± 0.13**	*0.26* ± *0.12*	2106
nsp31960	NSP_RS14115	CTB family bacteriocin	**1.08 ± 0.14**	**1.31 ± 0.11**	*0.28* ± *0.11*	2105
nsp31950	NSP_RS14110	CTB family bacteriocin	*0.77* ± *0.14*	**1.16 ± 0.11**	*0.08* ± *0.11*	2104
nsp49370	NSP_RS21715	Non-ribosomal peptide synthetase	*0.69* ± *0.11*	**1.06 ± 0.11**	*−0.36* ± *0.11*	3225
nsp49380	NSP_RS21720	Non-ribosomal peptide synthetase	*0.89* ± *0.13*	**1.81 ± 0.12**	*−0.59* ± *0.12*	3225
nsp49390	NSP_RS21725	2-isopropylmalate synthase	**1.01 ± 0.12**	**1.35 ± 0.13**	*−0.37* ± *0.14*	3225
**N_2_ fixation associated proteins**						
nsp12160	NSP_RS05470	Molybdate ABC transporter substrate-binding protein	**1.04 ± 0.35**	*0.53* ± *0.35*	*0.41* ± *0.41*	849
NA	NSP_RS06835	Mo-dependent nitrogenase C-terminal domain-containing protein	**1.08 ± 0.41**	**2.56 ± 0.17**	*−0.64* ± *0.46*	1054
nsp2880	NSP_RS01265	Nitrogenase	*0.56* ± *0.18*	**1.20 ± 0.14**	*−0.05* ± *0.16*	188
nsp36730	NSP_RS16250	HetP family heterocyst commitment protein	*0.34* ± *0.43*	*−0.37* ± *0.34*	**1.11 ± 0.40**	2416
nsp40520	NSP_RS17870	Putative nitrogen fixation protein NifT	*0.33* ± *0.27*	**1.34 ± 0.21**	*−0.99* ± *0.26*	2667
nsp40650	NSP_RS17920	Nitrogenase cofactor biosynthesis protein NifB	*0.95* ± *0.15*	**1.16 ± 0.10**	*−0.03* ± *0.13*	2674
nsp40800	NSP_RS17990	Nitrogenase molybdenum-iron protein subunit beta	*0.77* ± *0.22*	**1.34 ± 0.11**	*−0.29* ± *0.17*	2685
nsp40840	NSP_RS18010	Nitrogen fixation protein NifX	**1.10 ± 0.14**	*−0.21* ± *0.12*	*0.64* ± *0.14*	2687
nsp40850	NSP_RS18015	NifX-associated nitrogen fixation protein	**1.14 ± 0.13**	*−0.05* ± *0.11*	*0.57* ± *0.13*	2687
nsp40870	NSP_RS18025	Nitrogenase-stabilizing/protective protein NifW	**1.09 ± 0.17**	*−0.15* ± *0.17*	*0.59* ± *0.19*	2688
**Cell differentiation and metabolism**						
nsp46380	NSP_RS20405	ABC transporter permease DevC	*0.61* ± *0.25*	**1.21 ± 0.20**	*−0.24* ± *0.26*	3018
nsp8610	NSP_RS03865	Hormogonium polysaccharide biosynthesis protein HpsA	*0.54* ± *0.12*	**1.38 ± 0.14**	*0.44* ± *0.14*	590
nsp50440	NSP_RS22185	Glucose-6-phosphate dehydrogenase	**1.00 ± 0.12**	*0.92* ± *0.10*	*0.35* ± *0.12*	3290

Significantly differentially expressed genes were identified at an absolute log_2_-fold change ≥ 1 and a Benjamini–Hochberg adjusted *p*-value of ≤0.1. Log_2_-fold changes are given with standard error (*n* = 3) in bold (significant) or italics (non-significant change).

## 4. Discussion

Acclimation to long-term, harsh P limitation was studied in the strain *N. spumigena* CCY9414, which was isolated from a cyanobacterial summer bloom in the Baltic Sea and can thus serve as model for bloom-forming *Nodularia* spp. under such conditions (Stal et al., 2003; Voss et al., 2013). In contrast to our previous study (Hagemann et al., 2019), *Nodularia* CCY9414 was pre-cultivated two times for 10 days in P-containing medium, which resulted in a good P-status of the starting cultures. This assumption is supported by the low expression of P-regulated genes in cells grown for 7 days in P-replete conditions and the relatively high polyphosphate content in *Nodularia* CCY9414 filaments at the beginning of the experiment (Figure 1C). Despite the significant slower growth of cells under P-deplete conditions, these cultures were still able to triple their biomass during our experimental period of 3 weeks (Figure 1A). The question arises, where the necessary P is coming from, because the stored polyphosphate pool remained almost stable. The amount of available *o*-phosphate in the P-deplete medium was close to 1 μM, which is comparable to the values measured in the Baltic Sea during summer bloom events (Nausch et al., 2008). However, it has been shown that the high affinity Pst2 system in the model strain *Synechocystis* sp. PCC 6803 has an *o*-phosphate affinity of 0.07 μM (Pitt et al., 2010). Genes encoding proteins with high similarity to this Pst2 were highly up-regulated in *Nodularia* CCY9414 filaments grown under P-deplete conditions, hence, it can be assumed that this ABC transporter is still able to acquire *o*-phosphate even in the presumably P-free medium. It is also known that acclimation to P limitation induces other P-saving strategies such as reduction in the copy number of cellular DNA or a decrease in the proportion of phospholipids in cyanobacteria (van Mooy et al., 2009; Zerulla et al., 2016).

It remains unknown why *Nodularia* CCY9414 is not consuming the stored polyphosphate after sudden transfer to P-deplete conditions at the beginning of the experiment. In contrast, when the cells gradually acclimated to P limitation in the P-replete medium after 14 days, then the polyphosphate pool was stepwise consumed. However, still a substantial amount of polyphosphate was kept in these cells after 3 weeks of incubation although the dissolved *o*-phosphate in the medium was utterly consumed (Figures 1B, C). Presumably, the immediate drop in P from normal availability to very low amounts transforms the cell into a state, in which the polyphosphate reserves are stabilized, whereas under slowly P-decreasing conditions it is at least partially consumed as reported from P-limited model organisms (Lawrence et al., 1998; Gómez-García et al., 2003). Interestingly, only few expression changes were observed for genes encoding proteins for polyphosphate synthesis or breakdown in *Nodularia* CCY9414 filaments under P-replete or P-deplete conditions (Supplementary Table 3). This finding suggests that polyphosphate accumulation is mostly regulated at post-transcriptional level in *Nodularia* CCY9414 and possibly other cyanobacteria, because related genes for polyphosphate accumulation are also not part of the P-regulon in *Synechocystis* sp. PCC 6803 (Suzuki et al., 2004). Comparatively high polyphosphate contents have been also reported from other filamentous cyanobacteria such as *Trichodesmium* spp. under low external P concentrations in the environment (Orchard et al., 2010; Martin et al., 2014). It can be speculated whether the stable polyphosphate pool at low external P contents is saved to support the development the next *Nodularia* spp. generation or akinete formation, or whether it is a strategy to keep all the available phosphate in non- or slow-growing cells to minimize the amount potentially available for competing microorganisms in the microbial community.

In the unicellular model *Synechocystis* sp. PCC 6803, P-induced genes could be assigned to a P-regulon, because almost all P-induced genes are characterized by a defined P-box (YTTAAYYW NNN YTTAAYYW NNN YTTAAYYW) upstream of their promoters that is targeted by the P-sensing two component system PhoB/PhoR (SphB/SphR) (Suzuki et al., 2004). Here, we used RNA-seq differential gene expression analysis to define the P-stimulon in *Nodularia* CCY9414. Our attempts to find a similar, conserved P-box in front of homologous genes/operons in *Nodularia* CCY9414 were not successful. This result may indicate that the more diverse and larger P-stimulon is regulated by different transcriptional factors. The P-regulon of *Synechocystis* sp. PCC 6803 mostly contains genes for *o*-phosphate uptake, alkaline phosphatase and regulatory proteins for their expression (Suzuki et al., 2004), whereas *Nodularia* CCY9414 has a much wider capacity to acclimate to low P conditions as an adaptation to its ecological niche. In addition to *o*-phosphate, transporters and enzymes for the utilization of alternative P-sources are not only encoded in the genome but mostly also induced in cells shifted into P-deplete medium, which supports the view that they contribute to the growth of *Nodularia* spp. in brackish waters under P-limiting conditions. These include proteins for the utilization of phosphonates and phosphites as well as different external organic P-sources. Similarly, many genes for the uptake and utilization of different inorganic or organic P-sources have been up-regulated in *Trichodesmium* spp. occurring in regions of the Atlantic Ocean with increasingly limiting P availability (Cerdan-Garcia et al., 2022). Phosphonate utilization has recently been verified to sustain growth of marine cyanobacteria under different nutrient availability (e.g., Acker et al., 2022; Rabouille et al., 2022; Zhao et al., 2022). Interestingly, not all annotated transport systems are induced under our P-limiting conditions, for example the operon 1262 encoding for one probable phosphonate uptake system is rather down-regulated after 7-day growth under P-deplete conditions (Table 1). In the future, it would be interesting to study the response of this and other operons/genes for the metabolism of alternative P-sources at varying amounts of phosphonates or related compounds in the growth medium.

In addition to up-regulation of genes for proteins directly involved in P acclimation, many annotated genes from different functional categories were co-regulated with the P-stimulon in *Nodularia* CCY9414. This group included several genes, which are assumed to be of importance in the context of bloom events. In addition to genes for proteins in N_2_-fixation and gas vesicle formation, genes for bioactive compound synthesis were found to be overexpressed in filaments from P-deplete cultures (Table 2). *Nodularia* CCY9414 has many operons that are potentially capable to produce different toxins such as nodularin and other bioactive compounds (Voss et al., 2013). Such gene clusters are especially widespread in cyanobacterial strains known to form blooms, where they can play different functions, such as defense against grazers, intra- or extracellular info-chemicals within the population, or as allelopathic signals between different microorganisms in the scum of the bloom (Dittmann et al., 2013; Guljamow et al., 2021). Among them, one operon for the synthesis of a CTB family bacteriocin was identified, which was induced after 7 and 14 days of growth in P-deplete medium. Bacteriocins are known allelopathic compounds that inhibit growth of other (cyano)bacteria in microbial consortia (Flores and Wolk, 1986; Aharonovich and Sher, 2016). Bacteriocin synthesis clusters have not only been identified in the genomes of bloom-forming filamentous cyanobacteria, but are also present in the genomes of picocyanobacteria with smaller genomes, highlighting their importance for the organism because they are kept despite substantial genome reduction (Paz-Yepes et al., 2013). Recently, it has been shown that such strains occur very frequently in the waters of different salinities including the Baltic Sea in summer (Cabello-Yeves et al., 2022). Since most bloom formation associated genes, such as nitrogenase-related, gas-vesicle, bacteriocin and iron-acquisition genes, became more highly expressed only after 14 days under P-limiting conditions, we conclude that long-term harsh P-starvation can indeed be regarded as a potential trigger for bloom formation in *Nodularia* CCY9414 and likely other cyanobacteria.

## Data availability statement

The original contributions presented in this study are included in the article and the associated Supplementary material. Furthermore, the transcriptomic reads and processed feature counts are accessible from the GEO database (https://www.ncbi.nlm.nih.gov/geo/) with the following accession number: GSE213384. The scripts for bioinformatic sequence processing and statistical data analysis are available at http://doi.io-warnemuende.de/10.12754/misc-2022-0005. Data from all other measured parameters are available on PANGAEA (Santoro et al., 2022).

## Author contributions

MH, MS, and ML designed the study. MS performed the cyanobacterial cultivations, poly-P estimation, and RNA extractions. CH analyzed the RNA-seq data. MH and ML supervised the experiments. MH, MS, and CH evaluated the data. MH and MS wrote the manuscript that was reviewed by all co-authors. All authors contributed to the article and approved the submitted version.

## Footnotes

1
https://biocomputo.ibt.unam.mx/operon_mapper/

2
https://www.ncbi.nlm.nih.gov/geo/

3
https://services.healthtech.dtu.dk/service.php?TargetP-2.0
