# Supplementary material for: Acclimation of Nodularia spumigena CCY9414 to inorganic phosphate limitation – Identification of the P-limitation stimulon via RNA-seq
Source: Front Microbiol. 2023 Jan 4;13:1082763. doi: 10.3389/fmicb.2022.1082763 (PMC9846622; doi:10.3389/fmicb.2022.1082763)
Supplement: Supplementary file 2 [file Data_Sheet_2.PDF]

Supplementary information (SI) of the Frontiers in Microbiology manuscript

Acclimation of *Nodularia spumigena* CCY9414 to inorganic phosphate limitation - Identification of the P-limitation stimulon via RNA-seq

Running title: P-stimulon in *Nodularia*

Mariano Santoro[1,2]*, Christiane Hassenrück[1]*, Matthias Labrenz[1], Martin Hagemann[2]**

*The first two authors contributed equally to the study.

1 Environmental Microbiology, Department of Biological Oceanography, Leibniz-Institute for Baltic Sea Research Warnemünde (IOW), Rostock, Germany

2 Department of Plant Physiology, Institute for Biosciences, University of Rostock, Rostock, Germany

****Corresponding author:** Martin Hagemann, Institut für Biowissenschaften, Abteilung Pflanzenphysiologie, Universität Rostock, A.-Einstein-Str. 3, Rostock D-18059, Germany; Tel: +49(0)3814986110; Fax: +49(0)3814986112; Email: martin.hagemann@uni-rostock.de

SI Figure S1: Ammonium levels in cultures of *Nodularia* CCY9414.

SI Figure S2: **DAPI-staining for polyphosphate visualization in *Nodularia* CCY9414.**

SI Figure S3: Global changes of genes expression in cells of *Nodularia* CCY9414.

SI Table S1. General Linear Mixed Model (GLMM) for dry weights and polyphosphates.

SI Table S2. Post-Hoc analysis of the GLMM for dry weights and polyphosphate amounts.

SI Table S3: Assignment of phosphate-related gene expression changes to the P-regulated stimulon previously identified for *Nodularia spumigena* CCY9414 by Voss et al. (2013).

SI Data 1: Complete data set of evaluated gene expression changes in cells of *Nodularia* CCY9414 provided as separate excel file.

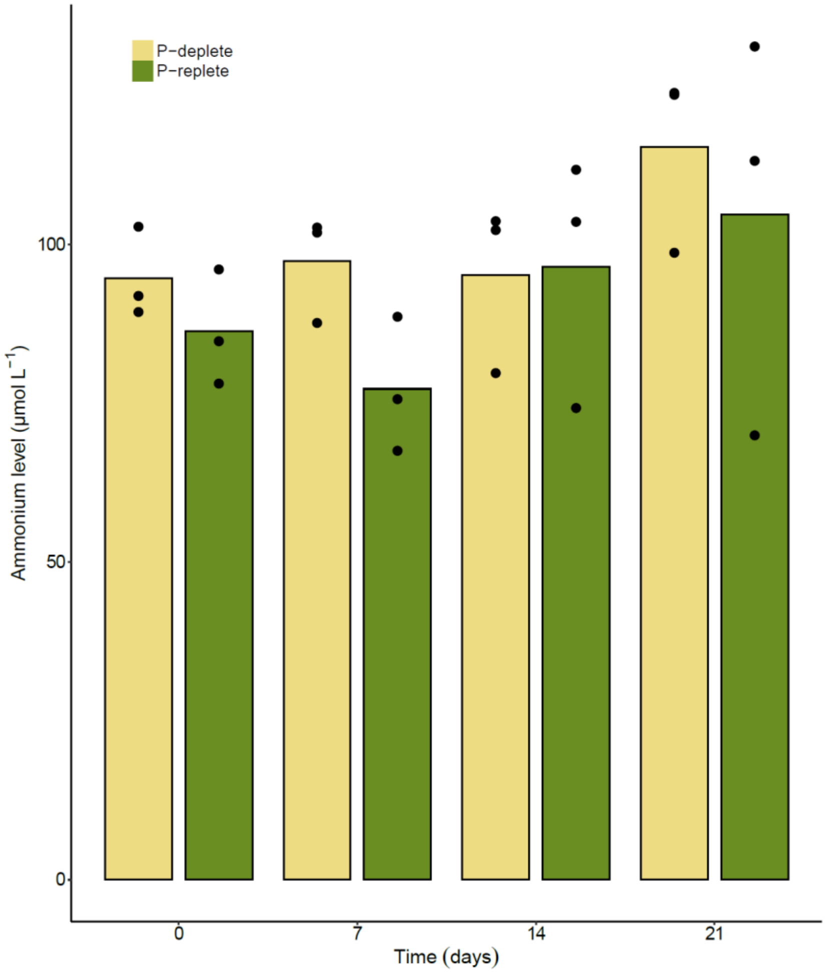

SI Figure S1: Ammonium levels in cultures of *Nodularia spumigena* CCY9414 under P-replete (+P) or P-deplete (-P) conditions. NH_4^+ was measured in the cultivation media for one of the experiments. Bars represent means and single data points indicate measurements

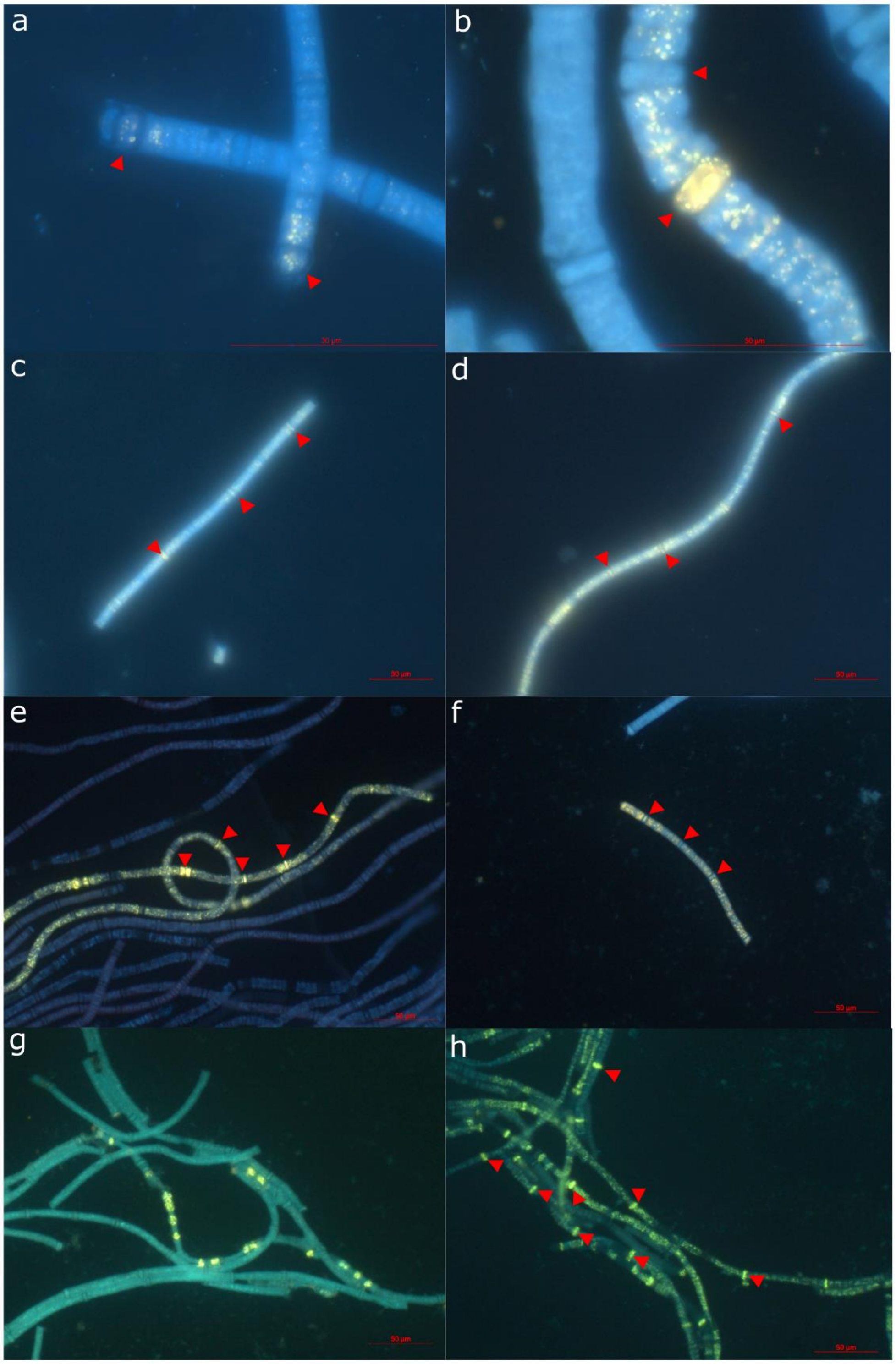

SI Figure S2: **DAPI-staining for polyphosphate visualization in filaments of *Nodularia spumigena* CCY9414** under P-replete (+P) or P-deplete (-P) conditions. Examples of filaments enriched with DAPI-stained polyphosphates are shown after 0, 7, 14 and 21 days in P-replete conditions (**a**, **c**, **e**, **g**) and in P-deplete conditions (**b**, **d**, **f**, **h**), respectively. Polyphosphate is accumulated in vegetative cells as well as in heterocysts (the latter are marked by red arrows).

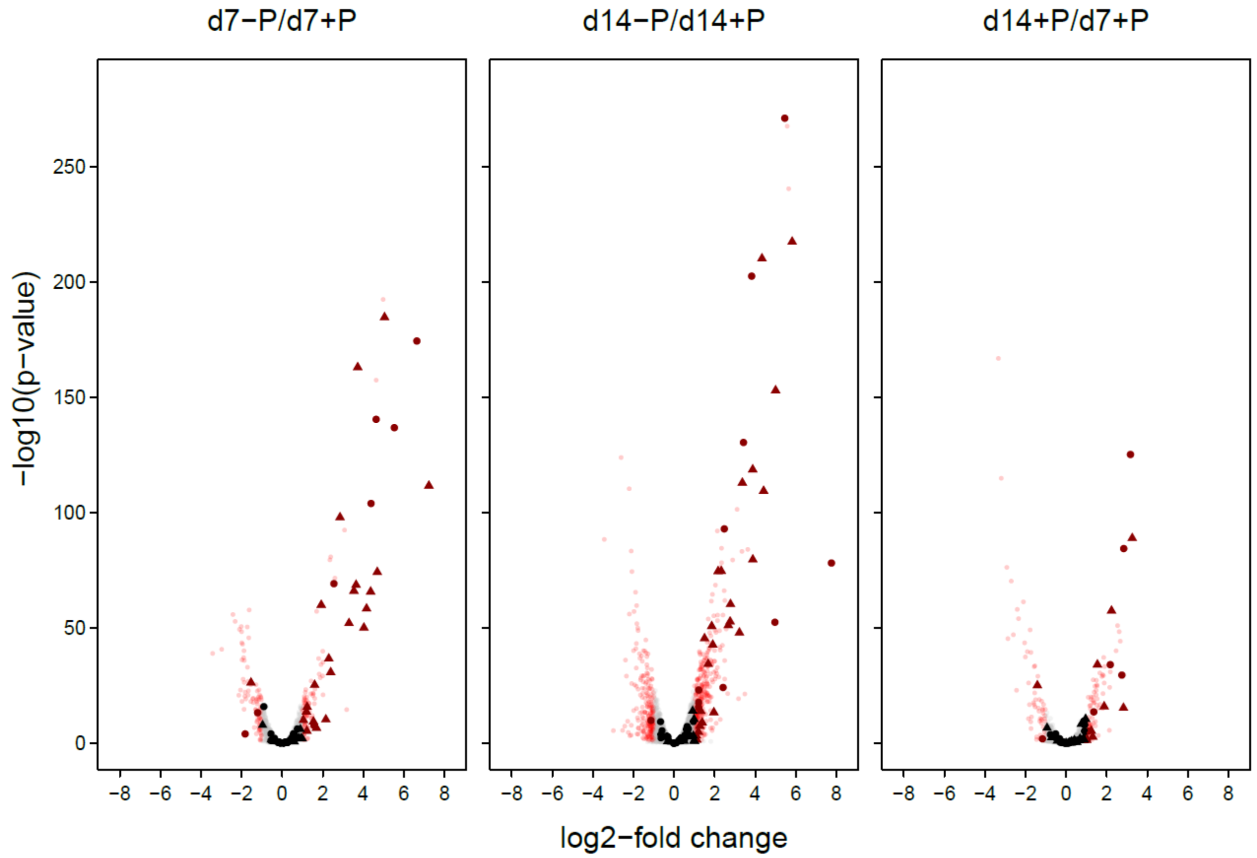

SI Figure S3: Global changes of genes expression in cells of *Nodularia* CCY9414 under P-replete (+P) or P-deplete (-P) conditions. Volcano plot of the result of the DESeq2 analysis. Genes encoding proteins possibly involved in P-acclimation according to Voss et al. (2013) are highlighted, while genes encoding annotated P transporters are shown as triangles.

SI Table S1. General Linear Mixed Model (GLMM) for dry weights and polyphosphates to test for differences between P conditions and sampling time points (**Figure 1, Figure 3**). DF = Degree of Freedom (numerator, denominator)

Predictor	Dry Weights			Polyphosphate amounts		
	**DF**	**F**	**p-value**	**DF**	**F**	**p-value**
P condition	1, 39	41.22	< 0.001	1, 37.04	30.44	< 0.001
Time point	3, 39	149.92	< 0.001	3, 37.04	1.19	0.091
P condition:Time point	3, 39	4.48	0.009	3, 37.05	1.14	0.082

SI Table S2. Post-Hoc analysis of the GLMM for dry weights and polyphosphate amounts. (SI table 1). SE = standard error; DF = degrees of freedom. Lower- and upper-case letters indicate significant differences between sampling time points for P-deplete and P-replete conditions, respectively (Figure 1, Figure 3).

P condition	Day	Dry weights				Polyphosphate amounts			
		**EMM**	**SE**	**DF**	**group**	**EMM**	**SE**	**DF**	**group**
P-deplete	0	0.298	0.028	12.6	a	2.545	0.395	5.44	a
P-replete	0	0.313	0.028	12.6	A	2.002	0.462	9.06	A
P-deplete	7	0.412	0.028	12.6	b	2.454	0.395	5.44	a
P-replete	7	0.512	0.028	12.6	B	1.278	0.395	5.44	AB
P-deplete	14	0.488	0.028	12.6	b	2.522	0.395	5.44	a
P-replete	14	0.656	0.028	12.6	C	0.880	0.395	5.44	AB
P-deplete	21	0.740	0.028	12.6	c	2.410	0.395	5.44	a
P-replete	21	0.921	0.028	12.6	D	0.744	0.395	5.44	B

SI Table S3: Assignment of phosphate-related gene expression changes to the P-regulated stimulon previously identified for *Nodularia spumigena* CCY9414 by Voss et al. (2013) after 7 or 14 days of P-limitation as well as during growth for 14 days in P-replete medium. Significantly differentially expressed genes were identified at an absolute log2-fold change ≥ 1 and a Benjamini-Hochberg adjusted p-value of ≤ 0.1. Log2-fold changes are given with standard error (n=3) in bold (significant) or italics (non-significant change). Bold locus tag names form a very large P-regulated cluster on the Nodularia CCY9414 chromosome (Fig. 6).

Old locus tag	New locus tag	Annotation	Operon	7d-P/7d+P	14d-P/14d+P	14d+P/7d+P
		**Inorganic P transport**				
nsp1550	NSP_RS00665	low affinity P permease	100	*0.25 ± 0.24*	*-0.01 ± 0.23*	*0.16 ± 0.26*
nsp16870	NSP_RS07580	low affinity P permease	1167	*-0.38 ± 0.12*	*-0.37 ± 0.11*	*-0.14 ± 0.12*
nsp15300	NSP_RS06870	freshwater SphX (P binding protein, similar to sll0540)	1058	*0.39 ± 0.15*	*0.10 ± 0.14*	*0.42 ± 0.16*
nsp28900	NSP_RS12745	periplasmic P binding protein PstS (similar to slr1247, high affinity, low velocity Pst2 system in 6803)	1909	**7.08 ± 0.13**	**4.33 ± 0.14**	**3.26 ± 0.16**
nsp28910	NSP_RS12750	PstC component of high affinity ABC P transporter	1909	**5.05 ± 0.17**	**3.36 ± 0.15**	**1.87 ± 0.21**
nsp28920	NSP_RS12755	PstA component of high affinity ABC P transporter	1909	**4.16 ± 0.25**	**3.22 ± 0.22**	**1.31 ± 0.34**
nsp28930	NSP_RS12760	PstB component of high affinity ABC P transporter ATP-binding protein component	1909	**4.03 ± 0.26**	**3.88 ± 0.20**	*0.89 ± 0.31*
nsp28940	NSP_RS12765	PstB component of high affinity ABC P transporter ATP-binding protein component	1910	*0.64 ± 0.22*	*0.52 ± 0.20*	*-0.21 ± 0.24*
nsp52600	NSP_RS23150	periplasmic P binding protein PstS (similar to sll0680, low affinity, high velocity Pst1 system in 6803)	3425	**3.72 ± 0.14**	**1.85 ± 0.12**	**2.24 ± 0.14**
nsp52610	NSP_RS23155	PstC component of high affinity ABC P transporter	3425	**2.85 ± 0.13**	**2.16 ± 0.12**	**1.54 ± 0.12**
nsp52620	NSP_RS23160	PstA component of high affinity ABC P transporter	3425	**2.30 ± 0.18**	**2.32 ± 0.12**	*0.96 ± 0.13*

nsp52630	NSP_RS23165	PstB component of high affinity ABC P transporter ATP-binding protein component	3425	**1.93 ± 0.12**	**1.50 ± 0.10**	*0.75 ± 0.12*
		**Phosphonate transport**				
**nsp7590**	NSP_RS03415	PhnF component of a C-P lyase, transcriptional regulator	516	*-0.35 ± 0.18*	*-0.32 ± 0.20*	*-0.43 ± 0.19*
**nsp7580**	NSP_RS03410	PhnG component of a C-P lyase	515	**1.54 ± 0.23**	**1.38 ± 0.22**	*0.66 ± 0.27*
**nsp7570**	NSP_RS03405	PhnH component of a C-P lyase	515	**2.15 ± 0.31**	**1.96 ± 0.25**	**1.01 ± 0.38**
**nsp7560**	NSP_RS03400	PhnI component of a C-P lyase	515	*0.90 ± 0.17*	*0.98 ± 0.14*	*0.41 ± 0.18*
**nsp7540**	NSP_RS03395	PhnJ component of a C-P lyase	515	*0.92 ± 0.29*	*0.94 ± 0.26*	*0.45 ± 0.32*
**nsp7530**	NSP_RS03390	PhnK component of a C-P lyase	515	**1.22 ± 0.24**	*0.79 ± 0.23*	*0.98 ± 0.28*
**nsp7520**	NSP_RS03385	PhnL component of a C-P lyase	515	*0.59 ± 0.34*	**1.10 ± 0.30**	*0.24 ± 0.37*
**nsp7510**	NSP_RS03380	PhnM component of a C-P lyase	515	*0.82 ± 0.25*	**1.27 ± 0.22**	*0.32 ± 0.29*
**nsp7500**	NSP_RS03375	hypothetical protein in *phn* cluster	515	*0.99 ± 0.32*	*0.49 ± 0.29*	*0.52 ± 0.37*
**nsp7490**	NSP_RS03370	hypothetical protein in *phn* cluster	514	*1.30 ± 0.48*	*0.97 ± 0.51*	*0.31 ± 0.63*
**nsp7480**	NSP_RS03365	PhnD component of phosphonate ABC transporter phosphate-binding periplasmic component	513	**7.23 ± 0.32**	**5.81 ± 0.18**	**2.82 ± 0.33**
**nsp7470**	NSP_RS03360	PhnC Phosphonate ABC transporter ATP-binding protein	513	**4.69 ± 0.25**	**5.00 ± 0.19**	*0.91 ± 0.29*
**nsp7460**	NSP_RS03355	PhnE Phosphonate ABC transporter permease protein	513	**4.36 ± 0.25**	**4.41 ± 0.20**	*0.97 ± 0.29*
**nsp7450**	NSP_RS03350	PhnE3 Phosphonate ABC transporter permease protein	513	**3.64 ± 0.20**	**3.87 ± 0.16**	*0.70 ± 0.23*
nsp35120	NSP_RS15575	PhnC1 Phosphonate ABC transporter ATP-binding protein	2314	**3.54 ± 0.20**	**2.67 ± 0.17**	*0.83 ± 0.27*
nsp35130	NSP_RS15580	PhnD1 Phosphonate ABC transporter phosphate-binding periplasmic component	2314	**1.05 ± 0.15**	*0.91 ± 0.11*	*0.26 ± 0.14*

nsp35140	NSP_RS15585	PhnE1 Phosphonate ABC transporter permease protein	2314	**1.20 ± 0.15**	**1.31 ± 0.16**	*0.00 ± 0.18*
nsp35150	NSP_RS15590	PhnH *(*truncated version, translationally coupled to *nsp35160 – phnM* component of a C-P lyase)	2314	*1.43 ± 0.45*	**1.12 ± 0.49**	*0.14 ± 0.59*
nsp18360	NSP_RS08220	PhnD2 Phosphonate ABC transporter phosphate-binding periplasmic component	1262	**-1.53 ± 0.14**	*0.43 ± 0.17*	**-1.42 ± 0.13**
nsp18370	NSP_RS08225	PhnC2 Phosphonate ABC transporter ATP-binding protein	1262	*-0.96 ± 0.16*	*0.26 ± 0.16*	*-0.94 ± 0.16*
nsp18380	NSP_RS08230	PhnE2 Phosphonate ABC transporter permease protein	1262	*-0.53 ± 0.20*	*0.37 ± 0.18*	*-0.72 ± 0.20*
		**Phosphite transport**				
nsp35050	NSP_RS15540	PtxA Phosphite ABC transporter permease protein	2311	**3.29 ± 0.21**	**2.78 ± 0.17**	**1.24 ± 0.24**
nsp35060	NSP_RS15545	PtxB Phosphite ABC transporter phosphate-binding periplasmic component	2311	**2.39 ± 0.20**	**2.76 ± 0.18**	*0.68 ± 0.22*
nsp35070	NSP_RS15550	PtxC Phosphite ABC transporter permease protein	2311	**1.60 ± 0.15**	**1.90 ± 0.13**	*0.40 ± 0.17*
nsp35080	NSP_RS15555	phosphite dehydrogenase, 2-hydroxyacid dehydrogenase	2311	**1.23 ± 0.14**	**1.68 ± 0.13**	*0.15 ± 0.16*
nsp35090	NSP_RS15560	LysR transcriptional regulator	2312	**1.69 ± 0.30**	**1.20 ± 0.26**	*0.88 ± 0.35*
		**glycerol-3-phosphate transport**				
nsp7940	NSP_RS03570	glycerol-3-phosphate ATP-binding protein component (*ugpC*) (no other components of the *ugp* operon present)	539	*0.14 ± 0.14*	*-0.35 ± 0.10*	*0.23 ± 0.13*
		**P stress inducible**				
nsp8220	NSP_RS03700	PhoH, phosphate starvation-inducible protein, predicted ATPase	561	*-0.13 ± 0.14*	*-0.01 ± 0.12*	*0.25 ± 0.14*
		**P storage and degradation of P polymers**				
nsp10230	NSP_RS04600	Ppk, polyphosphate kinase (ppk)	706	*0.48 ± 0.14*	**1.21 ± 0.12**	*-0.11 ± 0.13*
nsp29750	NSP_RS13110	Ppa, inorganic pyrophosphatase (ppa)	1960	*-0.55 ± 0.12*	*-0.36 ± 0.10*	*-0.52 ± 0.11*

nsp42550	NSP_RS18760	Ppx, exopolyphosphatase (ppx)	2790	*0.07 ± 0.18*	*0.53 ± 0.14*	*-0.10 ± 0.15*
		**Degradation of organic P sources**				
nsp6490	NSP_RS02895	glycerophophoryl diester phosphodiesterase (phytase domain)	448	**4.63 ± 0.18**	**5.45 ± 0.15**	*0.90 ± 0.13*
nsp7010	NSP_RS03145	atypical alkaline phosphatase (esterase-like activity of phytase family protein)	481	**5.53 ± 0.22**	**3.82 ± 0.12**	**3.17 ± 0.13**
nsp7000	NSP_RS03140	Metallophosphoesterase	480	**4.38 ± 0.20**	**3.42 ± 0.14**	**2.18 ± 0.17**
nsp6990	NSP_RS03135	DUF4114 domain-containing protein	480	**2.55 ± 0.14**	**2.47 ± 0.12**	*0.90 ± 0.14*
nsp12860	NSP_RS05760	DedA-like phosphatase	899	*-0.90 ± 0,10*	*-0.36 ± 0.10*	*-0.32 ± 0.10*
nsp12920	NSP_RS05785	alkaline phosphatase, extracellular	904	**6.64 ± 0.23**	**7.75 ± 0.41**	**1.07 ± 0.21**
nsp12930	NSP_RS05790	cation diffusion facilitator family transporter	904	**7.14 ± 0.16**	**4.97 ± 0.32**	**2.74 ± 0.23**
nsp12940	NSP_RS05800	PhoX-like phosphatase	905	**7.02 ± 0.17**	**5.41 ± 0.14**	**2.84 ± 0.14**
nsp18960	NSP_RS08485	putative PhoX phosphatase, DUF839 domain-containing protein	1304	**6.28 ± 0.15**	**5.69 ± 0.15**	**1.36 ± 0.17**
nsp20770	NSP_RS09270	COG4246 superfamily (esterase or phytase like)	1425	*-0.07 ± 0.21*	*-0.65 ± 0.21*	*0.35 ± 0.21*
nsp29340	NSP_RS12935	Metallophophoesterase	1934	**-1.82 ± 0.41**	*-0.67 ± 0.10*	**-1.16 ± 0.37**
nsp29350	NSP_RS12940	Metallophosphoesterase	1934	*-0.10 ± 0.15*	*0.00 ± 0.12*	*-0.06 ± 0.15*
nsp33000	NSP_RS14590	predicted phosphatase, HAD type	2178	*-0.09 ± 0.23*	*0.74 ± 0.19*	*-0.30 ± 0.22*
nsp31680	NSP_RS13985	metal dependent PHP family phosphoesterase	2090	*-0.56 ± 0.25*	*-0.66 ± 0.16*	*0.90 ± 0.17*
nsp35720	NSP_RS15805	acid phosphatase, metallophosphoesterase	2348	*0.25 ± 0.16*	*-0.04 ± 0.15*	*0.38 ± 0.17*
nsp46480	NSP_RS20445	metallophosphoesterase (GlpQ-like)	3023	*0.17 ± 0.14*	*-0.28 ± 0.14*	*-0.48 ± 0.15*
nsp53310	NSP_RS23475	alkaline phosphatase D family protein	3474	**5.39 ± 0.13**	**5.42 ± 0.14**	*0.91 ± 0.15*
		**Arsenate-related gene orthologs/operons**				
nsp40	NSP_RS00015	ArsA	2	*0.61 ± 0.14*	*0.94 ± 0.14*	*0.12 ± 0.15*

nsp1880	NSP_RS00820	ArsA	117	*-0.15 ± 0.10*	*0.26 ± 0.11*	*-0.35 ± 0.11*
nsp15480	NSP_RS06940	ArsA family ATPase	1069	*0.56 ± 0.12*	*0.64 ± 0.11*	*-0.08 ± 0.12*
nsp15490	NSP_RS06945	AAA family ATPase	1069	*0.32 ± 0.11*	**1.12 ± 0.13**	*-0.46 ± 0.12*
nsp33490	NSP_RS14830	ArsR (regulator of arsenate resistance)	2213	**-1.21 ± 0.15**	**-1.14 ± 0.17**	*-0.78 ± 0.18*
nsp33500	NSP_RS14835	SphX periplasmic P binding component of P ABC transporter	2213	*0.75 ± 0.14*	**1.21 ± 0.13**	*-0.22 ± 0.15*
nsp33510	NSP_RS14840	ArsJ associated glyceraldehyde-3-phosphate DH	2214	**1.57 ± 0.26**	**2.41 ± 0.23**	*0.09 ± 0.29*
nsp33520	NSP_RS14845	ArsJ, major facilitator superfamily permease	2214	*0.29 ± 0.20*	**1.20 ± 0.14**	*-0.25 ± 0.17*
nsp33540	NSP_RS14850	Acr3 (ArsB) Arsenical-resistance protein ACR3	2216	*0.07 ± 0.16*	*0.32 ± 0.13*	*0.08 ± 0.15*
nsp33550	NSP_RS14855	ArsH Arsenic-resistance protein	2216	*-0.21 ± 0.19*	*-0.26 ± 0.15*	*-0.01 ± 0.19*
nsp41360	NSP_RS18230	Likely ArsC, LLM class flavin-dependent oxidoreductase	2710	*0.21 ± 0.15*	*0.34 ± 0.12*	*-0.36 ± 0.13*
		**Haloacid dehalogenase-like hydrolases**				
nsp1610	NSP_RS00685	HAD-superfamily hydrolase	104	*0.24 ± 0.27*	*0.11 ± 0.19*	*0.63 ± 0.22*
nsp3740	NSP_RS01650	HAD-superfamily hydrolase	248	*0.07 ± 0.14*	*0.24 ± 0.12*	*-0.02 ± 0.14*
nsp6980	NSP_RS03130	HAD-superfamily hydrolase	479	*0.53 ± 0.15*	*0.71 ± 0.13*	*0.12 ± 0.16*
nsp48160	NSP_RS21195	glycoside hydrolase/HAD-superfamily hydrolase	3144	*-0.35 ± 0.15*	*-0.60 ± 0.12*	*0.28 ± 0.14*
		**P sensing and regulation**				
nsp10800	NSP_RS04850	PhoB (SphR) Response regulator	748	*0.16 ± 0.12*	*0.60 ± 0.12*	*-0.35 ± 0.12*
nsp10810	NSP_RS04855	PhoR (SphS) sensor kinase	748	*0.09 ± 0.11*	*0.34 ± 0.11*	*-0.19 ± 0.12*
nsp10830	NSP_RS04860	PhoU putative negative regulator of the Pi regulon	749	*-0.18 ± 0.14*	*-0.31 ± 0.09*	*0.17 ± 0.11*
